# The Association of Body Mass Index with COVID-19 Complications and Survival Rate at a Tertiary Hospital

**DOI:** 10.3390/life13071572

**Published:** 2023-07-16

**Authors:** Salma AlBahrani, Thekra N. Al-Maqati, Yaser A. Al Naam, Jaber S. Alqahtani, Abdullah S. Alqahtani, Saad AlRabeeah, Abdulelah M. Aldhahir, Faisal Alkhalaf, Hind R. Alzuraiq, Maryam Hamad Alenezi, Amal Alzahrani, Mohanad Bakkar, Zainab Albahrani, Rawan M. Maawadh

**Affiliations:** 1 Internal Medicine Department, King Fahad Military Medical Complex, Dammam 34313, Saudi Arabia; 2 Department of Internal Medicine, College of Medicine, Imam Abdulrahman Bin Faisal University, Dammam 34313, Saudi Arabia; 3 Department of Clinical Laboratory Sciences, Prince Sultan Military College of Health Sciences, Dammam 31448, Saudi Arabia; yaser@psmchs.edu.sa (Y.A.A.N.);; 4 Department of Respiratory Care, Prince Sultan Military College of Health Sciences, Dammam 31448, Saudi Arabia; 5 Respiratory Therapy Department, Faculty of Applied Medical Sciences, Jazan University, Jazan 45142, Saudi Arabia; 6 Pharmacy Department, King Fahad Military Medical Complex, Dammam 34313, Saudi Arabia; 7 Medical Administration Department, King Fahad Military Medical Complex, Dammam 34313, Saudi Arabia; 8 Training Department, King Fahad Military Medical Complex, Dammam 34313, Saudi Arabia; 9 Internal Medicine Department, National Guard Hospital, Alhassa 31982, Saudi Arabia

**Keywords:** body mass index, COVID-19, COVID-19 complications, ICU

## Abstract

A high body mass index (BMI) is a known risk factor for coronavirus infection in hospitalized patients. Our study examined the association between BMI and complications and the survival rate among COVID-19 patients. This retrospective analysis used data from a tertiary hospital in the Eastern Region of Saudi Arabia during two waves of the COVID-19 pandemic. The study included 600 participants, with the majority being between 41 and 60 years old (41.3%) and men comprising 63.5% of the sample. Approximately 42.5% of patients were obese, and 31.3% were overweight. The results showed that BMI was significantly linked to respiratory diseases (*p* = 0.013); end-stage renal disease (*p* = 0.021); and cardiovascular disease (*p* = 0.003) but not diabetes mellitus (*p* = 0.064). Death occurred in 10.8% of patients; 33.8% were admitted to the ICU; 13.8% needed mechanical ventilation; and 60.7% had lung infiltration. Obese patients with oxygen saturation levels below 93% were 2.45 times more likely to require mechanical ventilation than those in the normal-weight group. Overweight and obese patients were also more likely to require mechanical ventilation than normal-weight patients, with odds ratios of 3.66 and 2.81, respectively. The BMI categorized was not associated with survival rate in COVID-19-hospitalized patients using Kaplan-Meier survival plots (*p* = 0.061). However, the BMI categorized was associated with survival rate in COVID-19 ICU patients (*p* < 0.001). In addition, the overweight showed a statistically significant higher hazard ratio of 2.22 (*p* = 0.01) compared to normal-weight patients using a Cox regression model. A high BMI was identified as an independent risk factor for reduced oxygen saturation (<93%), the need for mechanical ventilation, lung infiltration, mortality, and longer ICU stays in COVID-19 patients.

## 1. Introduction

Obesity and overweight have been identified as risk factors contributing to the development of severe illnesses that lead to hospitalization for COVID-19 infection [1]. However, the association may be subject to index event bias, a form of collider bias. Most existing research investigating this potential link has compared the outcomes of both obese and non-obese patients in the intensive care unit (ICU) [2]. Since severe COVID-19 cases and obesity might result in hospitalization, a link between the two may not reveal the full picture. A large population-based investigation that controlled for collider bias found that people with a body mass index of 30 kg/m^2^ and above had a greater risk of COVID-19 death than those with a BMI of 30 kg/m^2^ or lower [3]. In Saudi Arabia, the latest survey shows that 33.7% of the adult population is obese, while 68.2% are overweight [4]. The high prevalence of overweight and obesity in the country indicates a lack of or ineffective preventive measures. Early results from single or oligo-center studies suggest that obesity may be a major reason why COVID-19 patients require hospitalization and are at higher risk of mortality [5] as the COVID-19 pandemic continues to spread [6].

In addition, being overweight increases the risk of contracting respiratory illnesses such as the flu. Conversely, individuals with a normal weight are less likely to experience complications from influenza, such as hospitalization, mortality, extended stays, or the need for mechanical ventilation, compared to those with a higher body mass index (BMI) [7]. A study investigated the risk of death among patients admitted to the ICU and found that a high BMI was associated with an increased risk of death [8]. However, a recent study with 193 COVID-19 patients showed that BMI was unrelated to the survival rate [9]. Currently, few studies on large, diverse patient populations are widely generalizable concerning hospitalization due to COVID-19. Thus, it is unclear how body mass index (BMI) affects clinical outcomes for COVID-19 patients’ cardiovascular, respiratory, and other systems [10].

Older people are less likely to develop a critical case of COVID-19 than younger people, but it is unclear whether being overweight benefits from the purported protective impact of youth [11]. Preliminary research suggests that young COVID-19 hospital patients who are overweight are at an increased risk of poor outcomes [12], but it has not been thoroughly investigated whether obesity and age might interact in a way that varies by age. Ignoring this possibility could have negative consequences for overweight young people and COVID-19 prevention measures [10]. Meanwhile, numerous meta-analyses and comprehensive reviews have found that severe acute respiratory infection outcomes appear to be worse in obese individuals [13]. However, most subjects in these studies were hospitalized after experiencing severe COVID-19 symptoms [14], which could indicate collider bias.

In a previous population study, researchers focused on the effects of obesity (defined as a BMI of 30 kg/m^2^ or more). However, little research has been conducted on how being overweight (defined as a BMI of >25 kg/m^2^) affects most people who do not have obesity [14]. Currently, no recent studies have firmly examined the association between BMI and complications and survival rate among hospitalized COVID-19 patients during two waves of the COVID-19 pandemic.

## 2. Materials and Methods

### 2.1. Study Design

The data related to demographic characteristics, health outcomes, and comorbidities used in this retrospective study were extracted from existing medical records of patients treated during the first two waves of the COVID-19 pandemic at a tertiary hospital in the Kingdom of Saudi Arabia from March 2020 to December 2021. Before conducting this research, approval was obtained from the Institutional Review Board of the Armed Forces hospitals in the Eastern Province (IRB #AFHER-IRB-2020-034). The data gathered from medical records consisted of 813 patients who tested positive for SARS-CoV-2 using RT-PCR procedures and were admitted to healthcare facilities. From the collected sample, 213 patients were excluded because 189 were missing data related to demographic characteristics, health outcomes, or comorbidities; ten were under 18 years old; and 14 were not included due to being underweight according to their BMI.

### 2.2. BMI Calculation

BMI is calculated by dividing a person’s weight in kilograms by the square of their height in meters. This measurement is critical for identifying obesity and overweight trends within the population, as it measures body fat. The formula for calculating BMI is as follows: BMI (in kilograms per square meter) = weight (in kilograms) / height^2^ (in meters).

### 2.3. Statistical Analyses

The raw data extracted from medical records were first compiled and organized in Microsoft Excel. Missing values were removed, and the data were then exported to SPSS v.26 for analysis. Descriptive and inferential statistical analyses were conducted to understand trends in the dataset. Nonparametric Kruskal-Wallis and Chi-square tests were used for independent continuous and categorical variables, respectively, to determine their relationship with BMI unadjusted data and COVID-19 outcomes, as well as other comorbidities. Covariates included in the multivariate logistic regression model were adjusted after being found statistically significant (*p* < 0.05) in the univariate analysis. The World Health Organization (WHO) BMI guidelines were followed in this study to facilitate comparison with previous research. The standard BMI grouping categorized normal weight as 18.5–24.9 kg/m^2^, overweight as 25–29.9 kg/m^2^, and obese as ≥30 kg/m^2^, with normal weight as the reference group.

The Kaplan-Meier estimate compared survival rates in three BMI categories: 18.5–24.9 kg/m^2^ for normal weight, 25–29.9 kg/m^2^ for overweight, and ≥30 kg/m^2^ for obese. Data were analyzed using the Cox regression model, adjusting for age, gender, cardiovascular disease (CVD), renal disease (RD), and end-stage renal disease (ESRD) to assess the hazard ratio (HR). The main outcome variable was death, and BMI was considered independent.

## 3. Results

### 3.1. Demographic Characteristics of the Study Sample

The demographic characteristics of the study sample showed that most participants (41.3%) were in the age range of 41–60 years, and 63.5% were male. The BMI distribution revealed that 42.5% of the participants were obese, 31.3% were overweight, and 26.16% had a normal weight. Regarding comorbidities, 47.5% of the participants had diabetes mellitus, 18.3% had cardiovascular disease, and 12% had respiratory diseases. Among patient admissions, 10.8% resulted in deaths, 33.8% were ICU admissions, 51.2% of patients had an oxygen saturation of 95% or less, 13.8% were on mechanical ventilation, and 60.7% had lung infiltration. The findings suggest that the data sample was diverse, enabling comparisons between different BMI groups to understand COVID-19 outcomes, including categorical data on age, gender, and various comorbidities, which reduced bias in the statistical analysis. Additionally, continuous data on the length of hospital stay (with a mean of 29 days) helped in understanding how BMI affects the severity of COVID-19 illness.

### 3.2. Assessing the Relationship between Demographics, Comorbidities, COVID-19 Outcomes, and BMI Category

Table 1 summarizes the relationship between demographic characteristics, comorbidities, COVID-19 outcomes, and BMI categories. The Chi-square test results indicate that age group and gender were significantly associated with BMI categories, with *p*-values of <0.001 and 0.005, respectively. Specifically, 44.3% of patients in the 41–60 age group were obese, and significantly more men (56.5%) than women (43.5%) were obese. No significant association was found between diabetes mellitus and BMI categories (*p* = 0.064). However, the results show significant associations between BMI categories and respiratory, cardiovascular, and ESRDs, with *p*-values of 0.013, 0.003, and 0.021, respectively.

Regarding COVID-19 outcomes, obesity was associated with several adverse outcomes, as the category shows significantly lower oxygen saturation (<93%) (*p* = 0.002), significantly higher cases of lung infiltrate (*p* < 0.001), significantly higher demand for mechanical ventilation (*p* < 0.001), and significantly higher death rates (*p* < 0.001). However, there were no significant associations between BMI categories and ICU admissions (*p* = 0.418). The Kruskal-Wallis test for investigating the differences in the length of hospital stay between BMI categories shows statistically significant differences (χ^2^(2) = 19.79, *p* < 0.001). A post hoc test using the Dunn-Bonferroni method revealed that patients with normal weight had significantly shorter hospital stays than those with obesity (*p* < 0.001). However, there were no significant differences between the other BMI categories and the normal or underweight group regarding the length of hospital stay (Table 2).

### 3.3. Estimated Relationship between BMI Categories as The independent Variable with COVID-19 Complication Outcomes and Mortality in Hospitalized and ICU Patients

Table 3 presents the logistic regression model for BMI categories adjusted for age, gender, RD, ESRD, and cardiovascular disease to estimate the relationship between COVID-19 complications and weight-related categories, namely obese and overweight, with normal weight as the reference group. The findings show that obese patients were more likely to have low oxygen saturation (<93%) than the normal weight group (OR = 2.450; *p* < 0.001). Additionally, significantly higher rates of obese (OR = 2.815; *p* = 0.012) and overweight (OR = 3.666; *p* = 0.001) patients required mechanical ventilation compared to those in the normal weight category. There was no statistically significant difference in lung infiltrate cases between the overweight and normal weight groups (OR = 0.894; *p* = 0.664). However, a statistically significant correlation was found between the obese group and lung infiltrate cases compared to the normal weight group (OR = 3.384; *p* < 0.001).

**Figure 1:**
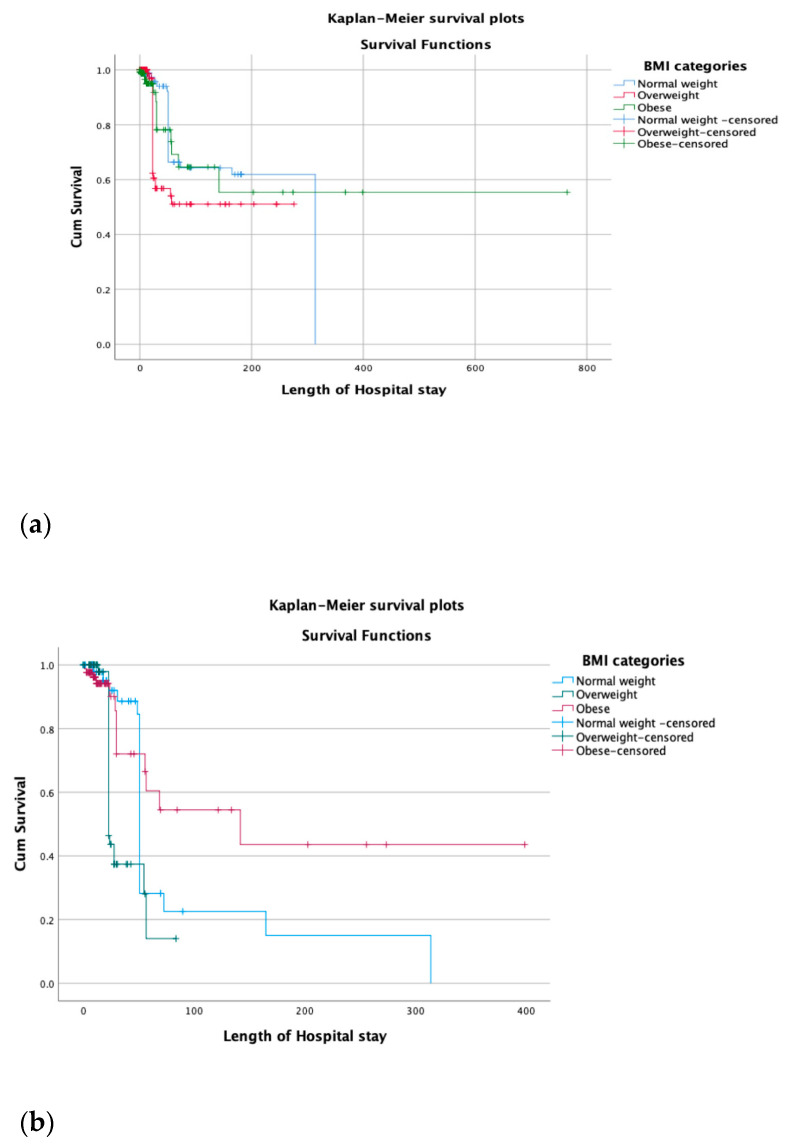
(**a**) Kaplan-Meier survival plots show the hospitalized COVID-19 patients’ survival based on different levels of BMI (less than 25, 25 to 30, more than 30) and show that the survival rate in these three levels of body mass index was not statistically significant (*p* = 0.71). (**b**) Comparison of ICU COVID-19 patients’ survival based on different levels of BMI was statistically significant. (*p* < 0.001).

**Figure 2:**
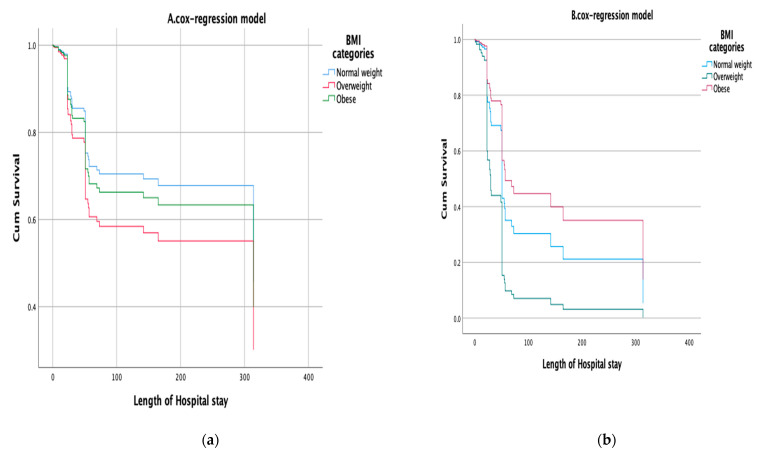
(**a**) Cox regression model to estimate HR and 95% CI of the impact of BMI on mortality of hospitalized COVID-19 patients, adjusting for potential confounder variables (age, gender, CD, RD, and ESRD). (**b**) Cox regression model to estimate HR and 95% CI of the impact of BMI on mortality of ICU COVID-19 patients, adjusting for potential confounder variables (age, gender, CD, RD, and ESRD).

Figure 1 illustrates the impact of BMI levels (18.5–24.9, 25–30, and >30) on the survival rate of hospitalized COVID-19 patients using Kaplan-Meier survival plots. The results indicate that the survival rate among these three BMI levels was not statistically significant (*p* = 0.061). However, for ICU COVID-19 patients, the survival rate was statistically significant (*p* < 0.001). The Cox regression model was used to estimate the hazard ratio (HR) for death as the main outcome variable. BMI was considered an independent variable, adjusting for age, gender, CVD, RD, and ESRD (Figure 2, Table 4). The model shows no statistical significance for the obese (HR = 1.175; *p* = 0.662) and overweight (OR = 1.535; *p* = 0.202) compared to the reference group among hospitalized patients. For ICU patients with COVID-19, the Cox regression model shows no statistical significance for the obese (HR = 0.675; *p* = 0.263), while the overweight group was statistically significant (OR = 2.222; *p* = 0.011) compared to the reference group (normal weight) (Figure 2, Table 4).

## 4. Discussion

A key finding was that among the patients admitted with COVID-19, 42.5% were obese and 31.3% were overweight. This result aligns with previous studies, including WHO [4], which reported that during the COVID-19 period, there was a prevalence rate of 68.2% for overweight and 33.7% for obesity in Saudi Arabia. Furthermore, other literature supports these findings, showing a high prevalence of obesity and overweight among COVID-19 patients in other countries, such as South Korea [5]. The finding can be explained by Lindgren et al., who indicate that obesity and overweight limit the respiratory capacity of individuals by reducing resting lung volumes [15]. In this respect, since COVID-19 is primarily a respiratory failure condition, patients who were overweight or obese were more likely to be affected. The high rates of obesity noted among COVID-19 patients were also linked to reduced access to healthy food and removed work environments and movement restrictions, which limited physical activity patterns [16,17]. In this respect, it was realized that increased obesity trends during the pandemic combined systemic challenges and poor personal decisions related to diet and physical exercise.

The results also revealed that BMI categories are not significantly linked to diabetes mellitus compared to ESRD, respiratory diseases, and cardiovascular disease, among the comorbidities. This finding suggests that obesity is unlikely to trigger insulin resistance and affect blood glucose absorption in cells. In keeping with our study, similar results were reported: no significant associations between BMI categories and diabetes [2]. The result contrasts with wider literature showing that there is a strong link between diabetes mellitus and obesity [18,19]. Essentially, studies have shown that obesity increases the development of insulin resistance by releasing more pro-inflammatory substances, thereby accelerating diabetes development [16]. The inconsistency between the obtained results and the literature needs further investigation. Therefore, addressing obesity should involve educating individuals on healthy nutrition as the primary strategy for avoiding unhealthy food intake, such as artificial sugars, which not only cause obesity but also contribute to diabetes mellitus.

When analyzing the relationship between BMI groups and COVID-19 outcomes, the study found a significant difference in mortality rates among the groups. Those with obesity also showed a significantly higher demand for mechanical ventilation, higher cases of lung infiltrate, and lower oxygen saturation compared to those in the normal weight categories. This finding suggests that obesity increases distress in patients due to its numerous comorbidities, which is consistent with Rietman et al.’s study [20], which found that obesity is strongly associated with physical frailty and negatively affects a patient’s ability to perform certain tasks. The study also reveals that a higher BMI increases the risk of adverse COVID-19 treatment outcomes because affected patients must deal with many health issues that weaken the immune system.

The current study also found that BMI categories were associated with age group and gender with *p*-values of <0.001 and 0.005, respectively. Similarly, a recent study in Saudi Arabia reported *p*-values of <0.0001 and 0.021 for age group and gender, respectively [21]. Since these *p*-values are less than 0.05, it can be inferred that age group and gender were statistically significant in demonstrating the association of COVID-19 with the patient’s BMI.

Furthermore, the study found *p*-values of 0.003 for cardiovascular disease, 0.013 for respiratory diseases, and 0.021 for ESRD, while noting 0.064 for diabetes mellitus. The results suggested that except for diabetes mellitus, BMI significantly affected most comorbidities noted during COVID-19 and adversely affected the health of the patients. The result is consistent with that of Townsend et al., showing that during COVID-19, there were higher rates of hospitalization for patients who were obese and had comorbidities such as cardiovascular diseases [22]. Similarly, the finding can be explained by Reitman et al., who showed that BMI was significantly related to physical frailty since obese individuals are more likely to suffer from damage to most body organs that are overworked [20]. The views explain the significant relationships with cardiovascular, respiratory, and ESRD diseases that are linked to specific organs but not diabetes mellitus.

Additionally, the study found a *p*-value of 0.418 for ICU admissions, *p* = 0.002 for oxygen saturation of 95% or less, <0.001 for mechanical ventilation, <0.001 for lung infiltrate, <0.001 for the length of hospital stay, and 0.004 for deaths. In comparison, other studies have reported *p*-values of <0.0001 for ICU admissions, <0.010 for oxygen saturation of 95% or less, 0.35 for mechanical ventilation, <0.0001 for lung infiltrate, 0.001 for the length of hospital stay, and 0.05 for deaths [22]. Since the *p*-values for these outcomes are less than 0.05, it is clear that patients’ BMIs are statistically significantly associated with COVID-19 outcomes in hospitalized patients. The study’s findings on deaths demonstrate the influence of patients’ BMI since the *p*-value is greater than 0.05.

The study also used a logistic regression model that adjusted for age, gender, RD, CVD, and ESRD to estimate the relationship between outcomes and weight-related categories, with normal weight as the reference group. The *p*-value for ICU admissions attributed to overweight and obesity was not significant, in contrast to other studies that found a *p*-value of 0.014 for ICU admissions attributed to overweight and <0.0001 for obesity [23]. Comparing these findings reveals that ICU admissions are an appropriate intervention for overweight and obese patients since their frequency in these groups is higher than in the reference group.

We found that the *p*-value for oxygen saturation of 95% or less attributed to being overweight was not significant, while the *p*-value for obesity was <0.001 with an OR of 2.450. A comparable study reported that oxygen saturation is less than 95% and statistically significant for overweight and obese COVID-19 patients, with *p*-values of <0.0001 and <0.0123, respectively [24]. The study’s findings estimated that overweight and obese patients were 3.666 and 2.815 times more likely to require mechanical ventilation than normal-weight patients, respectively. Wider literature has shown *p*-values of <0.019 for overweight and <0.0001 for obesity for mechanical ventilation [25,26,27,28,29,30].

Furthermore, we found that the *p*-value for lung infiltrate was not statistically significant for overweight compared to normal weight. In contrast, the *p*-value for lung infiltrate attributed to obesity was statistically significant, with a *p*-value of <0.001 and an OR of 3.384. Lung infiltrate attributed to overweight was 0.601, while it was <0.0001 for obesity [31,32,33]. These findings suggest lung infiltration is ineffective for overweight patients since its *p*-value is greater than 0.05. However, lung infiltration is 3.4 times more likely in obese patients than in the reference group.

We found that the survival rate of COVID-19 patients admitted to the hospital was not statistically significant (*p* = 0.71) based on different levels of BMI. However, the survival rate between BMI categories (*p* < 0.001) for those admitted to the ICU was statistically significant. These findings were similar to that of a large cohort study of Swedish ICU patients with COVID-19, where a high BMI was associated with an increased risk of death and a prolonged length of stay in the ICU [8]. In contrast, other studies have revealed that BMI is unrelated to the survival rate in COVID-19 patients admitted to the ICU [9].

## 5. Conclusions

This retrospective study was conducted during the first two waves of the COVID-19 pandemic, and a high BMI was identified as an independent risk factor associated with an increased risk of lower oxygen saturation (<93%), requiring mechanical ventilation, lung infiltrate, death, and prolonged length of stay in the ICU. Based on these findings, it is suggested that individuals with obesity should be closely monitored when hospitalized for COVID-19.

## Figures and Tables

**Table 1:** Relationship between demographics, comorbidities, and COVID-19 outcomes with BMI using the Chi-square test for the categorical variables (n = 600).

Variable	Total = 600	Normal Weight = 157	Overweight = 188	Obese 255	(*p*-Value)
(1) Demographical					
(a) Age group					<0.001 *
<20	26 (4.3%)	14 (8.9 %)	9 (4.8%)	3 (1.2%)	
20–40	158 (26.3%)	44 (28 %)	42 (22.3%)	72 (28.2%)	
41–60	248 (41.3%)	53 (33.8 %)	82 (43.6%)	113 (44.3%)	
61–80	145 (24.2%)	36 (22.9 %)	45 (23.9%)	64 (25.1%)	
>80	23 (3.8%)	10 (6.4 %)	10 (5.3%)	3 (1.2%)	
(b) Gender					0.005 *
Female	219 (36.5%)	50 (28%)	64 (34 %)	111 (43.5 %)	
Male	381 (63.5%)	132 (72 %)	123 (66%)	144 (56.5 %)	
(2) comorbidities	Total =600	Normal weight =157	Overweight =188	Obese 255	(*p*-Value)
(a) Diabetes mellitus					
Yes	285 (47.5%)	100 (55.4 %)	82 (43.6 %)	116 (45.5 %)	
No	315 (52.5%)	82 (44.6%)	105 (56.4%)	139 (54.5 %)	0.064
(b) Cardiovascular disease					
Yes	110 (18.3%)	26 (16.6 %)	49 (26.1%)	35 (13.7%)	
No	490 (81.7%)	131 (83.4 %)	139 (73.9%)	220 (86.3%)	0.003 *
(c)Respiratory Diseases (RD)					0.013 *
Yes	72 (12%)	27 (17.2%)	13 (6.9 %)	32 (12.5%)	
No	528 (88%)	130 (82.8 %)	175 (93.1 %)	223 (87.5%)	
End-Stage Renal Disease (ESRD)					0.021 *
Yes	33 (5.5%)	15 (5.1%)	4 (2.1 %)	14 (8.9%)	
NO	567 (94.5%)	240 (94.1 %)	184 (97.9 %)	143 (91.1%)	
(3) The outcomes	Total = 600	Normal weight = 157	Overweight = 188	Obese 255	(*p*-value)
(a) Admission to ICU					0.418
Yes	203 (33.8%)	48 (30.6 %)	70 (37.2 %)	85 (33.3 %)	
No	397 (66.16%)	109 (69.4 %)	118 (62.8%)	170 (66.7 %)	
(b) O2<93%					0.002 *
Yes	307 (51.2%)	68 (43.3 %)	87 (46.3%)	152 (59.6 %)	
NO	293 (48.8%)	89 (56.7 %)	101 (53.7%)	103 (40.4 %)	
(c) Mechanical ventilation					<0.001 *
Yes	83 (13.8%)	10 (6.4 %)	39 (20.7 %)	34 (13.3 %)	
No	517 (86.2%)	147 (93.6%)	149 (79.3 %)	221 (86.7 %)	
(d)lung infiltrate					<0.001 *
Yes	364 (60.7%)	85 (54.1 %)	90 (47.9%)	189 (74.1%)	
No	236 (39.3%)	72 (45.9 %)	98 (52.1%)	66 (25.9 %)	
(f) Death					0.004 *
Yes	65 (10.8%)	23 (14.6 %)	27 (14.4%)	15 (5.9%)	
No	535 (89.2%)	134 (85.4%)	161 (85.6 %)	240 (94.1%)	

* significant *p*-value < 0.05

**Table 2:** Kruskal-Wallis test investigating the length of hospital stay mean rank between the different BMI categories with post hoc analysis.

BMI	N = 600	Mean Rank	df	X^2^	*p*-Value	Post Hoc Analysis	*p*-Value
Normal weight 18.5–24.9	157	345.66	2	19.79	<0.001 *	Normal weight vs. Overweight	0.110
Overweight 25–29.9	188	306.56				Normal weight vs. Obese	<0.001 *
Obese ≥30 Kg/m^2^	255	268.23				Overweight vs. Obese	0.064

* significant *p*-value < 0.05

**Table 3:** Logistic regression model to estimate OR and 95% CI for the association between BMI categories and outcomes with potential confounders.

	Model 1. BMI Adjusted for Age, Gender, CVD, RD, and ESRD					
Variable	O2 < 93%		Mechanical Ventilation		Lung Infiltrate	
	OR (95% CI)	*p*-Value	OR (95% CI)	*p*-Value	OR(95% CI)	*p*-Value
Normal weight (reference group)	-	-	-	-	-	-
Overweight	1.55 (0.958–2.519)	0.074	3.666 (1.660–8.096)	0.001	0.894 (0.555–1.439)	0.664
Obese	2.450 (1.837–4.610)	<0.001	2.815 (1.261–6.285)	0.012	3.384 (2.111–5.477)	<0.001

**Table 4:** Cox regression model to estimate HR and 95% CI of the impact of BMI on mortality of hospitalized COVID-19 patients (A) and ICU patients (B) adjusting for potential confounder variables.

	Model A. BMI Adjusted for Age, Gender, CVD, RD, and ESRD			Model B. BMI Adjusted for Age, Gender, CVD, RD, and ESRD		
Variable	Death			Death		
	HR	(95% CI)	*p*-Value	HR	(95% CI)	*p*-Value
Normal weight (reference group)	-	-	-	-	-	-
Overweight	1.535	(0.794–2.966)	0.202	2.222	(1.204–4.098)	0.011
Obese	1.175	(0.570–2.421)	0.662	0.675	(0.339–1.345)	0.263

## Data Availability

The data presented in this study are available upon reasonable request from the corresponding author.
